# Correction: Podolska et al. Graphene Oxide Nanosheets for Localized Hyperthermia—Physicochemical Characterization, Biocompatibility, and Induction of Tumor Cell Death. *Cells* 2020, *9*, 776

**DOI:** 10.3390/cells13231927

**Published:** 2024-11-21

**Authors:** Malgorzata J. Podolska, Alexandre Barras, Christoph Alexiou, Benjamin Frey, Udo Gaipl, Rabah Boukherroub, Sabine Szunerits, Christina Janko, Luis E. Muñoz

**Affiliations:** 1 Department of Internal Medicine 3—Rheumatology and Immunology, Universitätsklinikum Erlangen, Friedrich-Alexander University (FAU) Erlangen-Nürnberg, 90154 Erlangen, Germany; gosiapodolskao2@wp.pl; 2 Univ. Lille, CNRS, Centrale Lille Univ. Polytechnique Hauts-de-France, UMR 8520-IEMN, F-59000 Lille, France; alexandre.barras@univ-lille.fr (A.B.); rabah.boukherroub@iemn.univ-lille1.fr (R.B.); sabine.szunerits@univ-lille1.fr (S.S.); 3 Department of Otorhinolaryngology, Head and Neck Surgery, Section of Experimental Oncology and Nanomedicine (SEON), Else Kröner-Fresenius-Stiftung-Professorship, Universitätsklinikum Erlangen, 91054 Erlangen, Germany; christoph.alexiou@uk-erlangen.de (C.A.); christina.janko@uk-erlangen.de (C.J.); 4 Department of Radiation Oncology, Universitätsklinikum Erlangen, Friedrich-Alexander-University (FAU) Erlangen-Nürnberg, 90154 Erlangen, Germany; benjamin.frey@uk-erlangen.de (B.F.); udo.gaipl@uk-erlangen.de (U.G.)

**Add New References**

In the original publication [1], there was a mistake in the legend for Figure 1C. Two new references should be added. The legend should contain a foot note as follows: “rGO-PEG was adapted from [12,13]”. The later references are changed accordingly. The correct legend and new references appear below.

**Figure 1:** Characterization of graphene oxide (GO), reduced graphene oxide (rGO), and PEGylated rGO (rGO-PEG): (**A**) UV/Vis absorption spectra of graphene derivatives. The optical absorption was measured in 1 cm quartz cuvette on UV/Vis spectrophotometer, and the wavelength range of 200–1000 nm was used. (**B**) FTIR absorption spectra of graphene derivatives. The optical absorbance was measured on a Nicolet 8700 FTIR instrument (resolution 4 cm^−1^). The signal recorded from a pure KBr pellet was used as background noise. (**C**) Representative transmission electron microscope (TEM) images of graphene derivatives. rGO-PEG was adapted from [12,13]. Scale bar, 100 nm. (**D**) Physicochemical characterization of graphene derivatives. (**E**) Representative photographs of graphene derivatives diluted in PBS or DMEM as depicted. (**F**) Determination of bacterial growth on Luria broth (LB) agar plates covered with 50 µg/mL of graphene derivatives. Positive control: *Escherichia coli* (strain DH5α), red arrow. Scale bar, 2 cm. (**G**) Quantitative measurement of bacterial endotoxin levels in graphene derivatives. UV/Vis, Ultraviolet-Visible; FTIR, Fourier-transform infrared spectroscopy; GO, graphene oxide; rGO, reduced graphene oxide; rGO-PEG, PEGylated reduced graphene oxide; LB, Luria broth; *E. coli*, *Escherichia coli*; EU, endotoxin units.

12. Turcheniuk, K.; Hage, C.H.; Heliot, L.; Railian, S.; Zaitsev, V.; Spadavecchia, J.; Boukherroub, R.; Szunerits, S. Infrared photothermal therapy with water soluble reduced graphene oxide: Shape, size and reduction degree effects. *Nano Life* **2015**, *5*, 1540002. https://doi.org/10.1142/S1793984415400024.
13. Turcheniuk, K.; Hage, C.H.; Spadavecchia, J.; Serrano, A.Y.; Larroulet, I.; Pesquera, A.; Zurutuza, A.; Pisfil, M.G.; Héliot, L.; Boukaert, J.; et al. Plasmonic photothermal destruction of uropathogenic *E. coli* with reduced graphene oxide and core/shell nanocomposites of gold nanorods/reduced graphene oxide. *J. Mater. Chem. B* **2015**, *3*, 375–386. https://doi.org/10.1039/c4tb01760a.

The authors state that the scientific conclusions are unaffected. This correction was approved by the Academic Editor. The original publication has also been updated.
